# Bats pre-adapt sensory acquisition according to target distance prior to takeoff even in the presence of closer background objects

**DOI:** 10.1038/s41598-017-00543-8

**Published:** 2017-03-28

**Authors:** Eran Amichai, Yossi Yovel

**Affiliations:** 1 Department of Zoology, Faculty of Life Sciences, Tel-Aviv University, Tel-Aviv, 6997801 Israel; 2 Sagol School of Neuroscience, Tel-Aviv University, Tel-Aviv, 6997801 Israel

## Abstract

Animals execute sensorimotor sequences to optimize performance of complex actions series. However, the sensory aspects of these sequences and their dynamic control are often poorly understood. We trained bats to fly to targets at different distances, and analysed their sensory behavior before and during flight to test whether they assess target distance before flight and how they adapt sensory acquisition in different situations. We demonstrate that bats’ sensory acquisition during approach-flight is more flexible than previously described. We identified acoustic parameters that illustrate that bats assess target distance before takeoff. We show that bats adapt their echolocation approach-sequences to target distance - ignoring closer background objects. At shorter distances, bats initiated their echolocation approach-sequence with distance-appropriate parameters, thus entering the approach sensory sequence “in step”. Our results suggest that in order to perform fine flight-manoeuvres, bats must maintain their sensorimotor plan in phase. To do this, they adapt acquisition according to target distance before initiating a complex sensory sequence based on a sensorimotor feedback-loop, even in complex acoustic environments, which impose other sensory reactions and restrictions. Though studying this in non-echolocating animals may prove difficult, such mechanisms are probably widely used in nature whenever complex series of sensorimotor actions are required.

## Introduction

Echolocating bats perceive the world acoustically by emitting sound signals and analyzing the returning echoes. Bats are renowned for their flexible active sensing^1–3^. For instance, it is well documented that flying bats adjust their echolocation signals based on the distance to nearby objects^3, 4^. However, it is currently unknown whether bats adapt their echolocation signals and sequences before takeoff according to the task they are about to perform, and if so, what is their adaptation strategy. When approaching a target, during an attack or towards landing, echolocating bats emit a highly stereotypic sequence of echolocation signals, often termed the ‘approach-phase’. This sequence begins with signals with specific acoustic parameters (e.g., durations and intervals) and it entails a gradual increase in emission rate and a gradual decrease in signal duration such that as the bat closes in on the target, it emits signals with well-defined acoustic characteristics at each distance from the target^1, 5–7^. Bats initiate this approach-phase sequence at a distance of 0.7–2.5 m from the target, and this distance depends upon the species and might change if the bat is attacking prey^8, 9^ or landing on an object^9–11^.

Several previous studies have examined bats’ flexibility in terminating the approach sequence^9, 12, 13^ but their flexibility when initiating it is yet to be studied. The success of the approach depends on emitting signals with appropriate distance dependent parameters. Since detection can occur at different distances, initiating the sequence at the “correct” values per detection distance to ensure sequence accuracy might be challenging. Moreover, it is unknown how bats deal with situations in which there are reflective objects closer than the target during the approach such as leaves or branches between the bat and its prey. Environments with nearby reflecting objects are usually referred to as cluttered environments and bats’ echolocation response to these situations are well described^1^. However, it is not clear if and how the clutter response affects the approach phase which is presumably guided by the distance of the target. We therefore set to examine three unstudied aspects of the echolocation approach behavior: (1) Do bats first assess distance to target and then take off or vice versa? (2) How do bats deal with situations in which they encounter background objects that are closer than the target when initiating the approach and during it, i.e., do they adapt their echolocation to the target or to the closer objects? (3) When distance to target is limited and the regular approach cannot be executed, do bats ‘compress’ their approach sequence, thus performing the complete approach over a shorter distance, or do they perform just part of the approach?

To test these questions, we designed an experiment that, in contrast to their usual behavior, forced the bats to initiate the approach-phase at different distances from the target and then to fly to it and land on it. This experimental paradigm allowed us to test whether bats assess the target’s location before take-off. Moreover, the bats had to do so in the presence of nearby background (non-target) objects that were closer than the target itself. The echolocation approach-phase is an example of a sensory program which accompanies a challenging motor task. Many animals probably practice similar sensory sequences, but bats’ reliance on active sound emission provides us with a unique opportunity to quantify the flexibility of such sensory behavior.

## Results

We trained four *Pipistrellus kuhlii* bats to takeoff from the same position – the roost wall (henceforth: ‘takeoff point’) in a flight box (200 × 50 × 50 cm^3^) and land on a target (an elevated platform with a 10 × 7 cm^2^ vertical landing plate) at a distance of 90, 140 or 190 cm (where mealworms where offered, see methods). Since the sensory approach phase in *P. kuhlii* in a large flight room usually starts 1.6 ± 0.4 m from the target (see methods), our experimental paradigm sometimes forced the bats to perform an approach that started at a shorter distance than usual. This allowed us to test their flexibility in initiating the approach, namely how they adapt the beginning of the sequence according to target’s distance. Moreover, since in this setup the bats were always within the approach range (2 m) they always started flying already in an approach mode in all distances. This allowed us to probe whether bats assess distance to target before takeoff.

**Figure 1:**
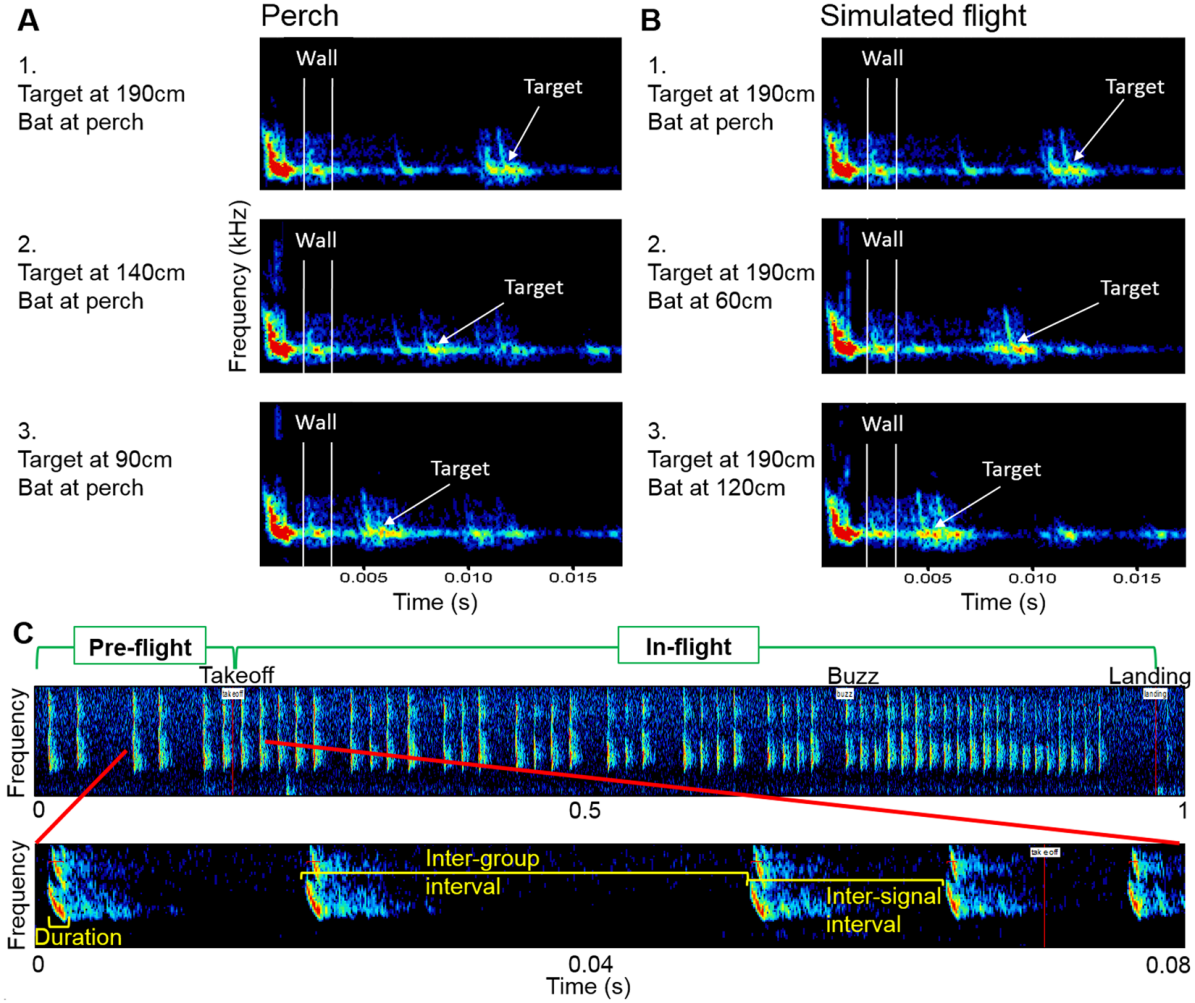
Background vs. target echoes and parameters for analysis. Ensonification of the target and the resulting echoes (**A,B**). Echoes from near background objects reached the bat after 1.7 ms, before those of the target. From the perch (takeoff point) (**A**), the echoes from the side walls (between white lines) reached the bat well before target echoes (marked by an arrow), whether the target is positioned at 190 cm (A1), 140 cm (A2) or 90 cm (A3). The same is true along a simulated flight path (**B**) to a target (positioned at 190 cm) from takeoff point (B1), after 60 cm of flight (B2) and after 120 cm of flight (B3). Though background echoes appear weaker than target echoes, this is a result of the higher directionality of the speaker compared to a bat’s echolocation beam (see methods). (**C**) Acoustic parameters used for analysis: Spectrogram of a typical flight’s echolocation sequence (top), beginning on the wall before takeoff. Notice the buzz- the terminal sequence of signals emitted right before contact with a target (the word **‘**Buzz**’** marks its initiation), before the landing (depicted at the end). Enlarged (bottom) is a segment containing the takeoff, illustrating the parameters analyzed in the study: signal duration, interval between signal groups (inter-group interval, used for pre-takeoff analysis) and interval between signals (inter-signal interval, used for in-flight analysis).

Note, that in all three target distances, the bat perching on its takeoff point in our setup first received echoes from the nearby neighboring walls of the box (right, left and ceiling) before the arrival of the target’s echo. We confirmed this by emitting *P. kuhlii* echolocation signals and recording the resulting echoes at several positions along the box (see methods). A bat echolocating from the takeoff point would receive echoes from the side walls as early as 1.7 ms after signal emission, long before the target echoes which would arrive at a delay of 5.9/8.2/11.1 ms for the 90/140/190 cm targets respectively (Fig. 1A,B). Moreover, the echoes from off-axis background objects were as loud as the target’s echo (due to their vicinity). The bats therefore had to assess the distance of the target while dealing with closer undesirable echoes. This situation was true during the majority of the flight until the bats were less than 30 cm from landing.

We used synchronized ultrasonic recordings and high-speed IR videography to analyze the echolocation and flight of the bats as they flew in complete darkness. We analyzed three echolocation parameters which characterize the approach sequence well: the intervals between consecutive signals, the intervals between groups of signals and the duration of the signals (see methods and Fig. 1C). We used videography to precisely localize each emitted signal and to document the bats’ movement.

### Bats assessed distance to target before takeoff

**Figure 2:**
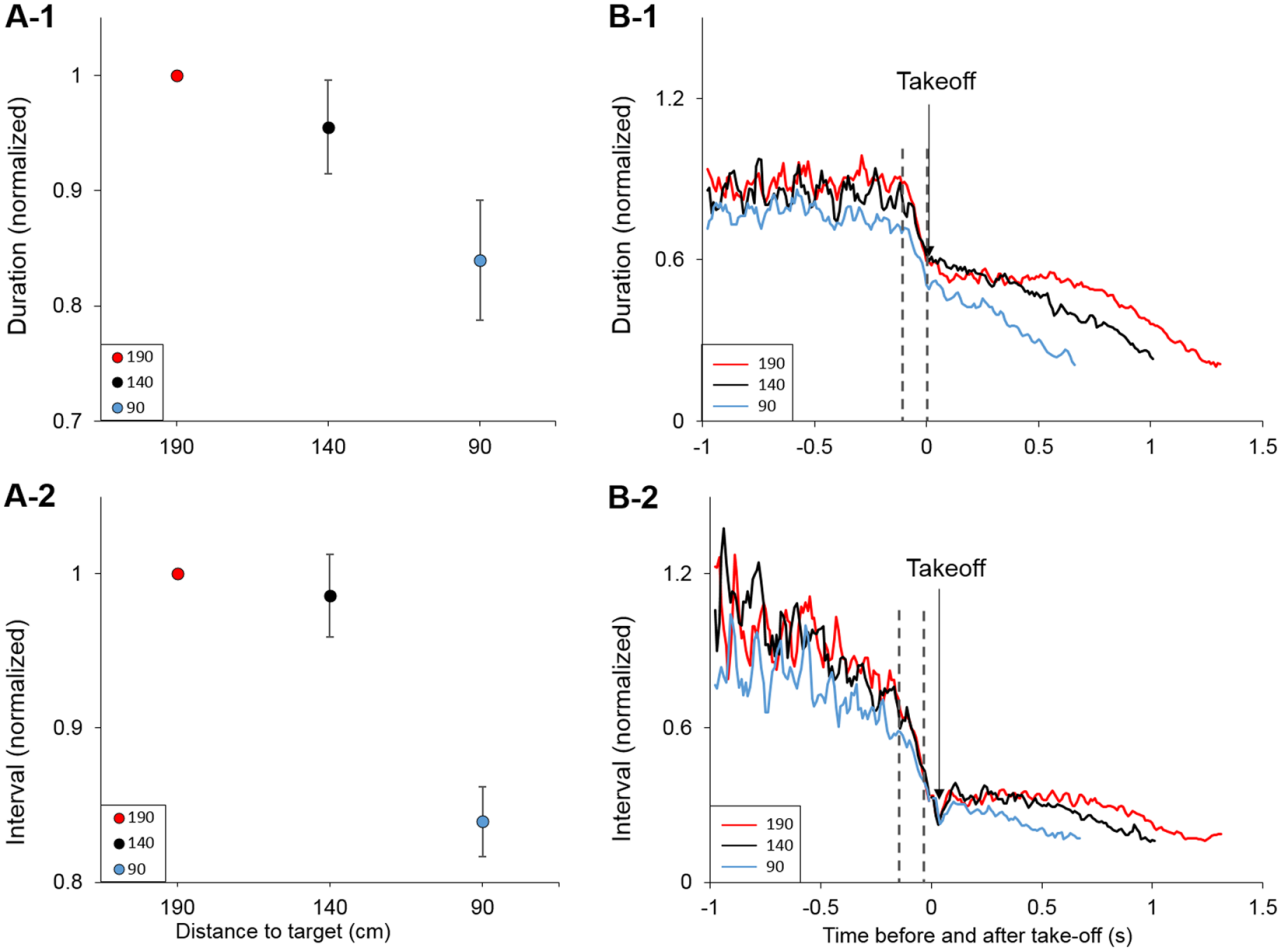
Bats assess distance to target before takeoff. (**A**) Echolocation signal duration (A-1) and interval between signal groups (A-2) are significantly shorter immediately before takeoff when the target is closer. Individual one-way ANOVA followed by Tukey’s post-hoc were performed for each individual for which values distribution was normal. For individuals whose values’ distribution was not normal we performed Kruskal-Wallis ANOVA on ranks followed by Dunn’s post-hoc. In all cases differences were significant between 190 cm and 90 cm. 140 cm was not always significantly different from both 90 and 190. For *duration*: Bat 1: H = 19.29, df = 2, P < 0.001 (*N* = 96[190 cm], 15[140 cm], 40[90 cm]). Bat 2: F = 6.77, df = 2, P = 0.002 (*N* = 19[190 cm], 32[140 cm], 32[90 cm]). Bat 3: F = 23.21, df = 2, P < 0.001 (*N* = 42[190 cm], 23[140 cm], 22[90 cm]). Bat 4: F = 14.75, df = 2, P < 0.001 (*N* = 118[190 cm], 65[140 cm], 76[90 cm]). For *intervals*: Bat 1: F = 4.44, df = 2, P = 0.017 (*N* = 12[190 cm], 6[140 cm], 34[90 cm]). Bat 2: H = 7.94, df = 2, P = 0.019 (*N* = 10[190 cm], 19[140 cm], 22[90 cm]). Bat 3: F = 7.04, df = 2, P = 0.03 (*N* = 18[190 cm], 11[140 cm], 12[90 cm]). Bat 4: H = 6.41, df = 2, P = 0.041 (*N* = 44[190 cm], 29[140 cm], 35[90 cm]). Statistical analyses were performed on raw individual data. Data were normalized for presentation (due to the differences in absolute individual values). We normalized by dividing each bat’s data by its 190 mean value (thus giving the mean at 190 an absolute value of 1 without variation, hence – no error bars). Data shown are the mean ± se for all bats. (**B**) Signal duration and intervals between signal groups as a function of time before and after takeoff (time point ‘0’). Longer signals and intervals are evident before takeoff and were probably used to assess the distance. Gray dashed lines mark the location in the sequence of the values shown in panels A-1 and 2. At takeoff there was a dramatic drop in these parameters. The mean of all bats is shown. See also Supplementary Figs S3 and S4.

Bats adapted the echolocation signals which they emitted immediately before takeoff according to the target’s distance proving that they assessed the target’s distance before takeoff rather than first fly and then adjust their sensory behavior (Fig. 2 and Supplementary Figs S3 and S4). Both the duration of the signals and the intervals between groups of signals (see Fig. 1C and experimental procedures) were significantly shorter the closer the target was at the moment of takeoff (Fig. 2A1–2, P < 0.05, F > 4/H > 6 in all bats, One-way ANOVA/Kruskal-Wallis ANOVA on ranks, see Fig. 2A and B for full details). As these two signal parameters were set by the bat before takeoff, they can therefore serve as proxies to the bat’s own perception of target distance. Moreover, bats adjusted their signals according to the distance of the target even though the first echo they received was always returning from the wall at a constant delay (of 1.7 ms) in all treatments (Fig. 1). This shows that bats segregate target from background when initiating the echolocation approach.

How did bats assess the target’s distance? Prior to each takeoff and while still stationary, bats emitted signals towards the target for a few seconds. During this period, bats used longer signals with longer intervals between them in comparison to the signals they emitted in flight. In flying bats, such echolocation sequences (i.e., with longer signals and longer intervals) are typical for the behavioral phase preceding the approach phase (Fig. 2B1–2). In our experiment, this stage of the behavior probably allowed the bats to reach an estimate of the distance to the target, such that they could takeoff while emitting the ‘right’ echolocation signals for this specific distance (see next paragraph).

### Bats adapted their echolocation to maintain a fixed approach phase

**Figure 3:**
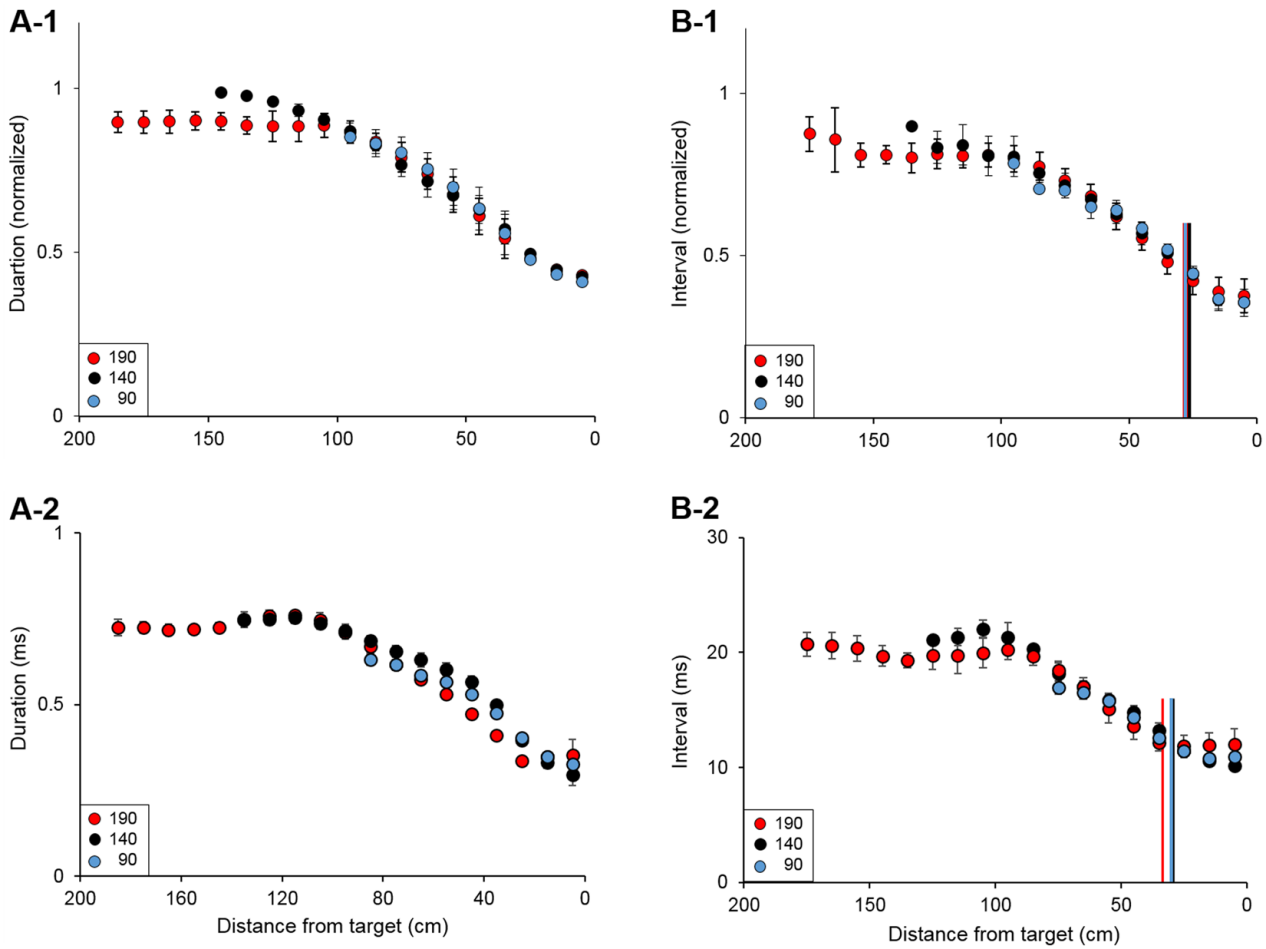
Bats maintained a fixed approach phase during flight. (**A**) When flying to a closer target (blue or black circles) the bats started their approach ‘in step’, meaning that signal duration at takeoff was appropriate as if the bat reached this distance at flight from a greater distance. Data are aligned to landing (distance ‘0’), shown are mean ± se, smoothed over a three-window period. A-1 All individuals, normalized data. A-2 An example of one individual. (**B**) The same strategy was evident with regards to emission rate and buzz initiation distance (vertical lines in panels B1-2, colours encoding is the same as the circles) – intervals between signals at the beginning of flight for a closer target were ‘in step’ as if the bat reached that distance from a farther starting point. Buzz was initiated at a fixed distance from target regardless of total flight distance. B-1 shows normalized data of all bats (normalization is the same as in Fig. A), B-2 is an example of one individual. See also Supplementary Figs S3 and S5.

From takeoff and until landing, bats emitted echolocation sequences that followed the approach-phase pattern, including a gradual reduction in signal duration and in the intervals between signals as the bat neared the target (Fig. 3). This was the case regardless of the initial distance to target (e.g., 90, 140 or 190 cm). Interestingly, when the bats flew shorter distances (e.g., 90 vs. 190 cm) and thus less time, they did not compress the entire approach sequence into this shorter time period. Instead, they started the approach ‘in step’ at the appropriate phase according to the distance to target, and thus they only performed the part of the approach from this distance and until the landing point. An analysis of the last 90 cm– the part of the flight overlapping in all conditions – showed that at any given distance from the target, bats used signal durations and inter-signal intervals that did not differ whether the takeoff was 190, 140, or 90 cm from the target. (Mixed model ANCOVA with takeoff distance and in-flight distance from target taken as factors and bat as random effect. *Signal duration* – df = 2, F = 1.89, p = 0.15. *Inter-signal intervals* – df = 2, F = 1.19, p = 0.3). Namely, a bat that took off at 90 cm from the target, emitted signals with a duration and at a rate as if it started at 190 cm and was now reaching the 90 cm point. The subsequent reduction in signal duration and intervals (between 90–0 cm) was done at the same rate in all conditions (the same was true for 140 cm, Fig. 3 and Supplementary Fig. S5).

The approach sequence ended with a ‘buzz’ – a terminal sequence of signals (typically emitted right before contact with a target) that is characterized by a constant maximum repetition rate and minimum signal duration. As part of their effort to maintain a fixed approach sensory sequence, bats also initiated this terminal sequence (the buzz) at a constant distance from the target regardless of the takeoff distance (See vertical lines in Fig. 3B and Supplementary Fig. S3). There was no significant difference in buzz initiation distance in any of the bats except for individual 4 whose buzzes in 140 cm were initiated 2 cm farther than in both the 90 cm and 190 cm treatments. One-way ANOVA/Kruskal-Wallis ANOVA on ranks, Bat 1: H = 2.29, df = 2, P = 0.318. Bat 2: F = 0.45, df = 2, P = 0.639. Bat 3: H = 2.14, df = 2, P = 0.343. Bat 4: H = 7.8, df = 2, P = 0.02).

### Bats adjusted their motor approach such that deceleration always started at the same distance from the target

**Figure 4:**
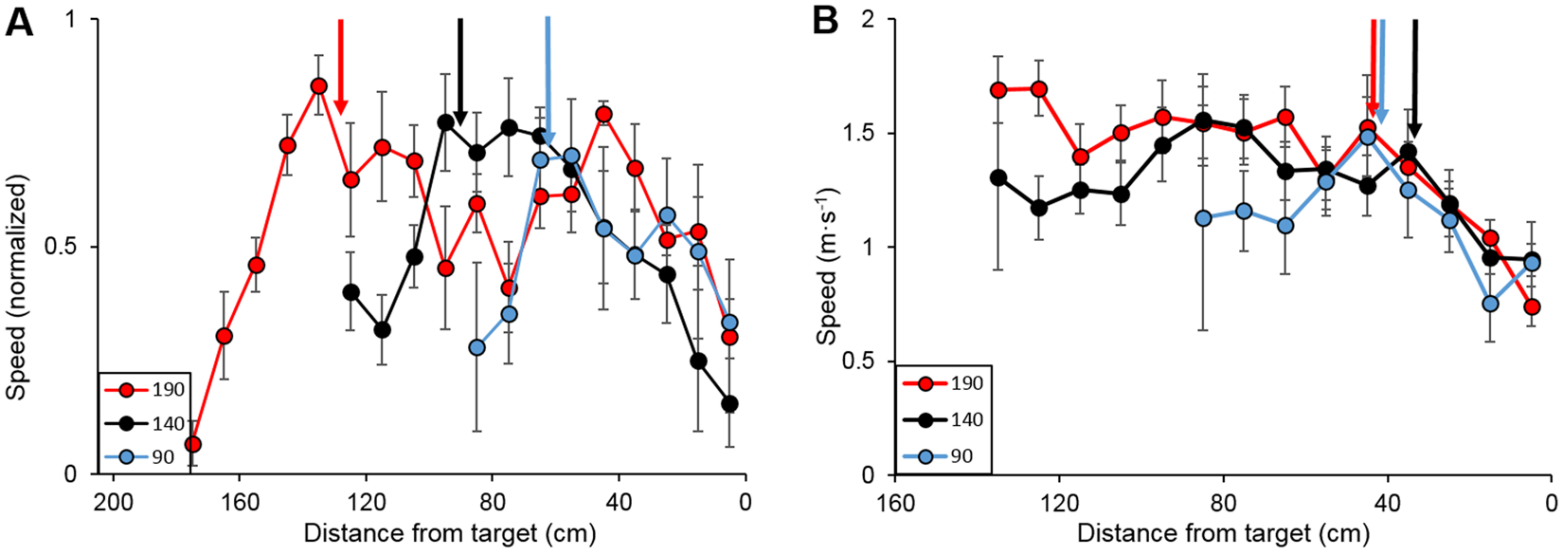
Bats reduce speed towards landing at a constant distance from target. Speed over distance to target for the 3 different takeoff distances. After initially increasing speed to reach a roughly stable ‘cruising’ speed (end of acceleration marked with correspondingly coloured arrows in panel A), the bats then decreased speed in preparation for landing. This was done at a constant distance from target (on average 50–60 cm), regardless of initial distance at takeoff (marked with arrows on panel B to simplify the presentation). Data are aligned to landing (distance ‘0’), shown are mean ± se. (**A**) Mean + se for all individuals. Data were normalized by dividing each bat’s average values for a given trial (target distance at takeoff) by the maximum value in that trial. (**B**) Non-normalized data from one individual. For two individuals the camera did not cover the first 30 cm of the flights to 190 cm.

Bats’ movement was similar, independently of the target’s distance at takeoff. At all flight distances (i.e., 90, 140 and 190 cm), the bats first gradually increased speed to reach a similar ‘cruising speed’, and they then reduced their speed at a roughly constant distance from the target (50–60 cm, Fig. 4) as preparation for landing. This behavior suggests that the bats’ motor plan aimed to maintain a fixed deceleration pattern at the final phase of the approach.

## Discussion

Echolocating bats have an inherent sensorimotor challenge: as flying mammals, they move fast, but they suffer from a short detection range resulting from the strong attenuation of high frequency echolocation in air^14, 15^. This combination could lead to missed feeding opportunities, as well as to collisions or non-optimal approaches to landing. To mitigate this problem, bats have evolved a highly-stereotypic sensory acquisition scheme (the echolocation approach-phase) which accompanies the motor approach and is executed when an approach (to attack or landing) begins. This sensory scheme includes a very accurate reduction in signal duration and a precise increase in emission rate as a function of the distance to the target. In brief, reducing the intervals between the signals increases information flow, while shortening the signals’ duration contributes to better ranging accuracy and to reducing potential ‘blind-zones’ which might result from an overlap between the emitted signal and the returning echo^6^. The approach phase has been thoroughly documented in many studies (e.g. refs. 9, 12 and 13) but bats’ flexibility in initiating it has hardly been tested. For a given species and a given task (e.g., landing), the approach behavior has been described as highly stereotypical, beginning at a certain distance and including a reduction of signal duration and signal-intervals from a specific initial value to a specific ending value. Here, we show that a bat can initiate its approach in the middle and still achieve smooth landing. More generally, we used the fact that bats adjust their signals in a task dependent manner as a tool to study flexibility in sensory acquisition. We were able to take advantage of this tool to investigate how bats optimize their sensory approach, finding that it is more dynamic than previously described.

*P. kuhlii* bats normally initiate the echolocation approach sequence at a distance of 1.6+/−0.4 m from the target. We therefore faced bats with a situation in which they had to begin their approach at shorter distances and examined their sensory flexibility. Such a scenario could occur in reality when a target is detected later than usual, for example in the case of a very weak target such as a mosquito (a typical prey of these bats) which suddenly appears in the acoustic field of view. Our findings indicate that bats pre-adapt their sensory plan before takeoff in order to maintain the precise coupling between the echolocation signal design and the motor plan which characterizes the approach sequence. The signal design at each distance therefore remained constant independent of the original takeoff distance (although it was affected by the acoustic environment; see below). To achieve this, before takeoff, bats first assessed the distance to the target by emitting longer signals with longer intervals between them (Fig. 2). Interestingly, bats already started adjusting the signal parameters when on the wall - note the decrease in inter-pulse-intervals before takeoff in Fig. 2B2. This implies that they were ‘tuning’ their signals in order to find the right parameters before takeoff. However, bats did not need to tune their echolocation signals to the final takeoff parameters – note the sudden drop in signal parameters (e.g., duration) at takeoff in Fig. 2B2–3. The bats repeated this behavior before every flight, even if they had recently performed a successful landing.

Importantly, in all conditions the perching bats did not adapt their echolocation signals to the closest objects, which were always the neighboring walls, but to a specific object - their landing target. How bats process complex acoustic environments with a multitude of echoes returning after every emitted signal has been the subject of some research. Surlykke *et al*.^16^ for example, showed that bats process echoes sequentially, while shifting their acoustic gaze to do so. Beetz *et al*.^17^ demonstrated a neural mechanism in the bat brain that suppresses late echoes thus always focusing sensory reception on the first echo returning from the nearest object. In our setup however, such a mechanism would have led to ill-processing of incoming information as the target of interest (i.e., the landing platform) was not the nearest object. Our analysis indeed shows that the bats were able to segregate the target’s echoes from early undesirable background echoes and to specifically adapt their signals to the target. This suggests that functional mechanisms may override this basic neuronal suppression.

Although the bats ignored the earlier wall echoes when assessing the distance to the target, these echoes did have a clear effect on the approach sequence. We compared signal duration at takeoff (Fig. 2A1) to the durations of signals emitted by a control group of four additional bats at the same distances. This group was trained to land on a platform in a large, uncluttered flight room (Supplementary Fig. S6, methods). Bats in our small multi-echo (i.e. highly cluttered) setup used significantly shorter signals than those flying in the uncluttered room. Shortening signal duration is a well-known response to clutter, but it has not been described in the context of the approach sequence because most experimental setups do not have objects that are closer to the bat than the target. Factoring peripheral environmental structure into the approach sequence design is yet another degree of freedom that bats are capable of controlling in order to optimize sensory acquisition.

Our findings thus contradict the general notion that echolocation signal parameters are deterministically defined by the distance of the target in two ways. (1) Bats emitted two different types of signals at the same distance from the target - one type of longer signals was used to assess target distance before takeoff, while the other type (of shorter signals) was emitted at the initiation of flight. (2) We show that the signal parameters of the approach sequence depend on the distance of the nearest clutter objects even when the distance of the target does not change.

Maintaining a fixed approach phase, as our bats exhibited, probably minimizes necessary ‘last minute’ adjustments in such a short distanced flight. In our setup, the bats were stationary and had ample time to assess the target’s distance before takeoff. It is an open question whether a flying bat that detects a target at a distance of less than 2 m, can rapidly assess its distance and execute the correct sensory approach response.

Although all bats applied the same sensory and motor adaptations (namely assessing the target’s distance and entering the approach phase in-step), they displayed some inter-individual variability in the absolute acoustic values of the echolocation signals that they emitted (Supplementary Fig. S7). For example, individual 2 consistently used longer intervals between its signals in comparison with the other bats, both to assess target distance when still on the wall (Supplementary Fig. S7A), and during flight (Supplementary Fig. S7B) although it did not seem to fly faster. This individual also initiated its buzz later than the others (Supplementary Fig. S7C). This behavior resulted in a total lower sampling rate of the environment, and perhaps also in less time for landing preparation. This reduced sensory acquisition however, was not translated into any visible impairment in its landing performance. The study of sensorimotor individuality is a topic that is mostly neglected by studies which report and compare the mean behaviors.

Sensorimotor feedback loops, as we demonstrate here in bat echolocation, are also important for other animals which rely on other sensory modalities, but they are very difficult to measure. The active nature of echolocation provides us with a unique opportunity to examine sensorimotor planning via an assessment of the echolocation signal parameters - their duration and the intervals between them in this study – allowing us to infer not only how the bat reacts to its environment, but what the environment appears like to the bat; The signals emitted by a bat can be used to investigate how it perceives its environment. This highlights the usefulness of species which rely on active sensory acquisition such as echolocating and electrolocating animals in the study of behavior and cognition.

## Materials and Methods

### Animals

Four adult female *Pipistrellus kuhlii* bats were captured under permit from the Israel Nature and Parks Authority, permit no. 2011/38137. Procedures were carried out under permit of the Institutional Animal Care and Use Committee operating according to the Israel Health Ministry, permit no. L-11-043. Bats were housed in the experimental setup (see below), with food and water available *ad libitum*. The bats were kept under a 16:8 h light:dark cycle, with a corresponding temperature cycle of 26:23 ± 2 °C.

### Experimental setup

Bats were trained to land on a target - an elevated platform where mealworms were offered (25 cm above the floor) in an elongated flight box (50 × 50 × 200 cm^3^, Supplementary Fig. S1). The bats were first trained to eat mealworms offered by hand while both bat and mealworm were held in a small metal plate. When the bats learned to grab and eat the mealworms on their own (after 2–4 days) the plate was left under the target for a period of 2–3 days. The next stage was to leave a second plate on the target and gradually decrease the amount of mealworms on the bottom plate. After an additional 3–4 days, when all bats were documented eating from the top plate, the bottom plate was eliminated and the training was complete. The walls and floor of the box were covered with echo-attenuating acoustic foam, while the ceiling was made of transparent acrylic to allow observation and videography. On one end of the box was a slit roost, and bats had to fly from the roost wall to the platform (which was 90, 140 or 190 cm away, 25 cm above ground) where mealworms were offered. The roost wall served in all cases as the takeoff point. Videography was done using two high-speed IR cameras (optitrack s250e, Natural Point Inc., USA), at a rate of 100 fps. Echolocation signals were recorded at a 250 kHz sampling rate using an omnidirectional ultrasonic microphone (Knowles with Avisoft Bioacoustics pre-amplifier) positioned under the platform and pointing towards the takeoff point. Recording was activated automatically by an intensity threshold-crossing trigger feature of the software (USGH, Avisoft Bioacoustics, Germany).

### Experiment design

During each session the target was positioned at a different distance from the takeoff wall: 90 cm, 140 cm, and 190 cm. The bats were left in the flight box (one bat at a time) for a period of 5–8 days for each distance (distance order was randomized and different for each bat). During this period the bats were kept in the box 24 hours a day, and they were free to fly to the platform whenever they wished, while we (video and audio) recorded their flights. We conducted all experiments via a remotely controlled computer to let the bats fly to the target at their own volition and without interference. Each bat was required to fly to all distances until it had performed at least ten flights to each distance. For a detailed table of total flights analyzed see Supplementary Table S2. The bats followed their natural activity pattern, so all flights were performed during the dark hours under complete darkness.

### Data extraction and analysis

We used Saslab Pro software (Avisoft Bioacoustics, Germany) to analyze the echolocation recordings. We analyzed the following parameters: (1) signal duration (peak −12 db (start) – −9 db (end)), (2) inter-signal-interval (time lapse from the start of one signal to the start of the following signal in a sequence, in flight) and (3) inter-group-interval (where a “group” was defined as two or more signals with inter-signal intervals between them which were shorter than the interval before and after the group). These parameters were analysed both on the wall before takeoff and in flight. For the pre-takeoff results we show the duration of the last signal on the wall and the time lapse between the last group of signals before takeoff and the next group of signals (which included the takeoff. Takeoff always occurred within group). We identified this parameter, together with the duration of the last signals on the wall, to be very good proxies of the bat’s perception of distance. For the flight results we show the signals’ duration and the interval between signals. We do not show the intervals between groups for the flight as there are only a few groups along the approach which makes this parameter very sparse. For a visualization of analyzed parameters see Fig. 1C. Parameters were measured automatically from the spectrogram (window size 128, overlap 87.5%). We visually inspected all analyzed signals.

We used Motive software (Natural Point Inc., USA) to analyze video recordings. We identified exact takeoff and landing times, and by synchronizing video to echolocation we could measure the distance from the target at which buzz was initiated. We used an in-house Matlab (Mathworks, Inc., USA) script to measure flight velocity.

To synchronize audio with video recordings we used a custom made audio-visual device that was connected to the pre-amplifier. This device was a millisecond-accurate digital clock with an IR visible LED display that upon triggering produced a light and an audible low-frequency sound pulse. We were thus able to locate each trigger event in both the spectrogram and the video, and align both recordings with an accuracy of 1–2 ms.

Statistical analyses were done using Sigmaplot software (Systat software Inc., USA). Charts were prepared in Excel software (Microsoft corp., USA) and Illustrator software (Adobe Systems, Ireland).

### Background echo recordings

To analyse the echoes from background objects, we ensonified the target from the bats’ takeoff point and from two additional points along their flight path (after 60 and 120 cm of flight), and recorded the returning echoes with a microphone positioned directly underneath the speaker. We repeated this procedure in a total of five situations: at the takeoff point with targets at 190, 140, and 90 cm, and in two in-flight points at 60 cm and 120 cm for the 190 cm target. At all points the speaker-microphone apparatus was located at mid height – 30 cm above ground, pointing directly towards the target. We created an artificial signal with similar spectro-temporal characteristics to the signals produced by the bats: a 1 ms FM sweep from 100 kHz to 35 kHz. We used an AD converter (116 H ultrasonic player, Avisoft Bioacoustics) at a sampling rate of 500,000 Hz and an ultrasonic speaker (Vifa, Avisoft Bioacoustics) and played-back the signal at an intensity level equal to that used by the bats (108 db SPL at 10 cm for the terminal frequency of 37 kHz). This speaker is much more directional than the bat’s echolocation beam, meaning that less energy was directed towards the side walls (the main background objects). Since the resulting background echoes were only ~5 db weaker than target echoes the real bat’s emissions would have probably resulted in background echoes which were at least as loud as ours.

### Un-cluttered flight room control recordings

We used data recorded from four additional individuals to compare signal duration between cluttered and un-cluttered environments. This control served to test whether environmental clutter affects approach phase echolocation parameters as it affects the echolocation search phase. We also used these sessions to measure the distance from the landing target at which the approach phase is initiated in this species.

Bats were trained to search for and land on an elevated (1.2 m) platform located at a randomly changing position in a large flight room (4 × 5 × 2.5 m^3^). Walls and ceiling were covered with echo-attenuating acoustic foam. All experiments were performed in complete darkness. An array of 16 tracking cameras (Raptor cameras with Cortex analysis software, Motion Analysis, USA) was evenly spread around the walls to cover an overlapping field of ~95% of the room to provide 3D flight paths and calculate distance from target. 22 ultrasonic microphones (Knowles with synchronized pre-amplifier, Avisoft Bioacoustics, Germany) were spread around the walls in three heights and synchronized to the tracking cameras recordings. We analysed signals from the best SNR channel (see methods in ref. 7) for the comparisons. Detailed sample sizes can be found in Supplementary Table S2.

### Ethics statement

Bats were captured under permit from the Israel Nature and Parks Authority, permit 2011/38137. Procedures were done under permit of the Institutional Animal Care and Use Committee operating according to the Israel Health Ministry, permit L-11-043.

## Electronic supplementary material

Supplementary Information
