# Supplementary material for: Bats pre-adapt sensory acquisition according to target distance prior to takeoff even in the presence of closer background objects
Source: Sci Rep. 2017 Mar 28;7:467. doi: 10.1038/s41598-017-00543-8 (PMC5428694; doi:10.1038/s41598-017-00543-8)
Supplement: Supplementary file 1 — Supplementary Information [file 41598_2017_543_MOESM1_ESM.pdf]

Bats pre-adapt sensory acquisition according to target distance prior to takeoff even in the presence of closer background objects

Eran Amichai[a] and Yossi Yovel[a,b, *]

Authors affiliations:

[a] Department of Zoology, Faculty of Life Sciences, Tel-Aviv University, Tel-Aviv 6997801, Israel

[b] Sagol School of Neuroscience, Tel-Aviv University, Tel-Aviv 6997801, Israel

[*] To whom correspondence should be addressed

Corresponding author: Yossi Yovel,

Department of Zoology, Faculty of Life Sciences, Tel-Aviv University,

Tel-Aviv 6997801, Israel.

Telephone: 972-506463642.

Email: yossiyovel@hotmail.com

Supplementary Information

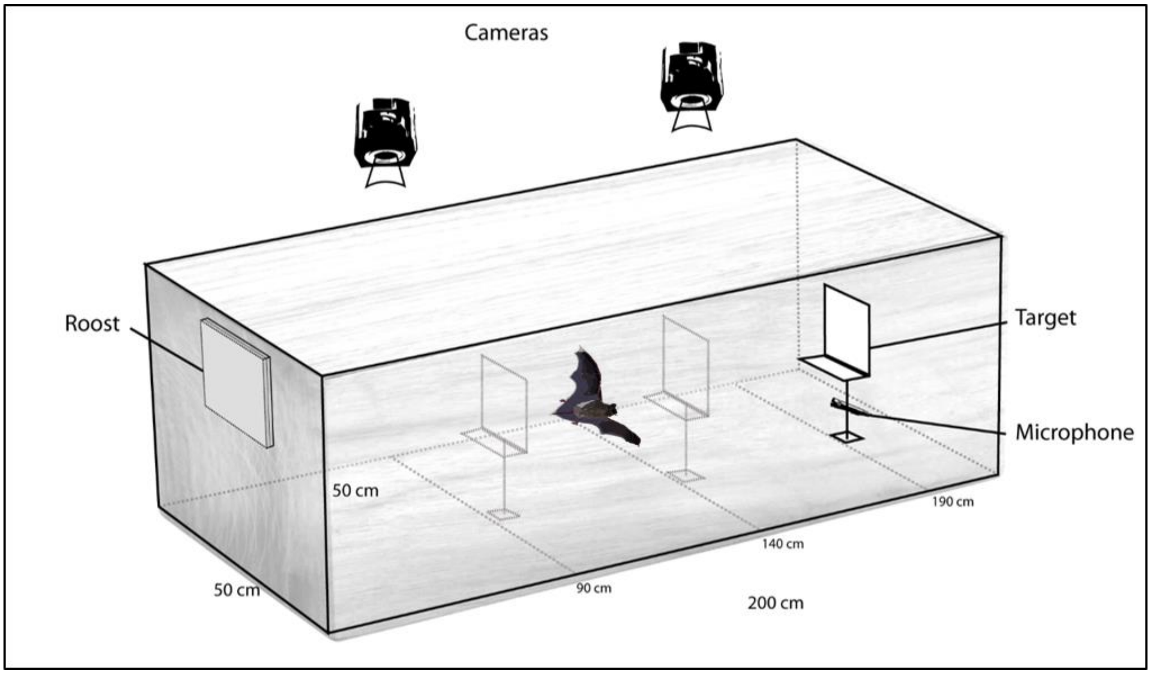

S1. Experimental setup. A layout of the experimental setup including: the flight box with the target (at different positions). The target was an elevated platform with a vertical landing plank, where mealworm were offered. Also shown are the ultrasonic microphone and high-speed IR cameras.

**1. Main experimental results – small (i.e. cluttered) flight chamber**												
**Distance**	**190cm**				**140cm**				**90cm**			
**Parameter** / **Individual**	N	Last *ISI* (ms)	Pre-flight *Dur* (ms)	Buzz initiation distance (cm)	N	Last *ISI* (ms)	Pre-flight *Dur* (ms)	Buzz initiation distance (cm)	N	Last *ISI* (ms)	Pre-flight *Dur* (ms)	Buzz initiation distance (cm)
**Bat 1**	34	45.3±1.5	1.3±0.02	31.6±1.7	6	48.1±3.3	1.3±0.06	31.2±3.9	12	37±2.3	1.2±0.02	34.4±2.2
**Bat 2**	22	57.96±2	1.4±0.04	23±0.7	19	53.9±2.1	1.27±0.03	24±0.7	10	45.6±3.7	1.2±0.04	23.6±1.1
**Bat 3**	12	52.6±2.5	1.7±0.08	33.6±1.4	11	53.1±1.5	1.4±0.06	29.1±2.1	18	44.5±1.6	1.1±0.05	30.2±2.2
**Bat 4**	35	50.2±2	0.87±0.02	23.7±0.9	29	47.2±1.5	0.9±0.02	21.3±0.6	44	45.6±3.7	0.82±0.01	24.5±0.8

**2. Uncluttered control – large flight room**							
**Distance**	**Approach beginning**	**190cm**		**140cm**		**90cm**	
**Parameter** / **Individual**	*Distance from target (cm)*	*N*	*Duration (ms)*	*N*	*Duration (ms)*	*N*	*Duration (ms)*
**Bat 1**	1570.9±103	7	2.9±0.05	7	1.9±0.16	10	0.99±0.04
**Bat 2**	1718.3±69	12	2.8±0.1	8	1.9±0.07	9	1.4±0.06
**Bat 3**	1714.3±73	6	3.01±0.05	8	2.4±0.07	12	1.3±0.08
**Bat 4**	1526±41	10	2.7±0.07	14	2.35±0.04	13	1.2±0.07

S2. Basic statystics of flights analyzed per distance per bat. The upper part of this table (part 1) regards the results of the main experiment described in this article, and lists the descriptive statistics of the flights to each distance target per bat. *N* is the number of flights performed to each distance. The lower part (part 2) lists the distance from target at which approach phase began and the number (*N*) of signals analyzed from bats flying in an un-cluttered, large flight room control, and the average durations of these signals at specific distances from the target. Values given are mean ± se.

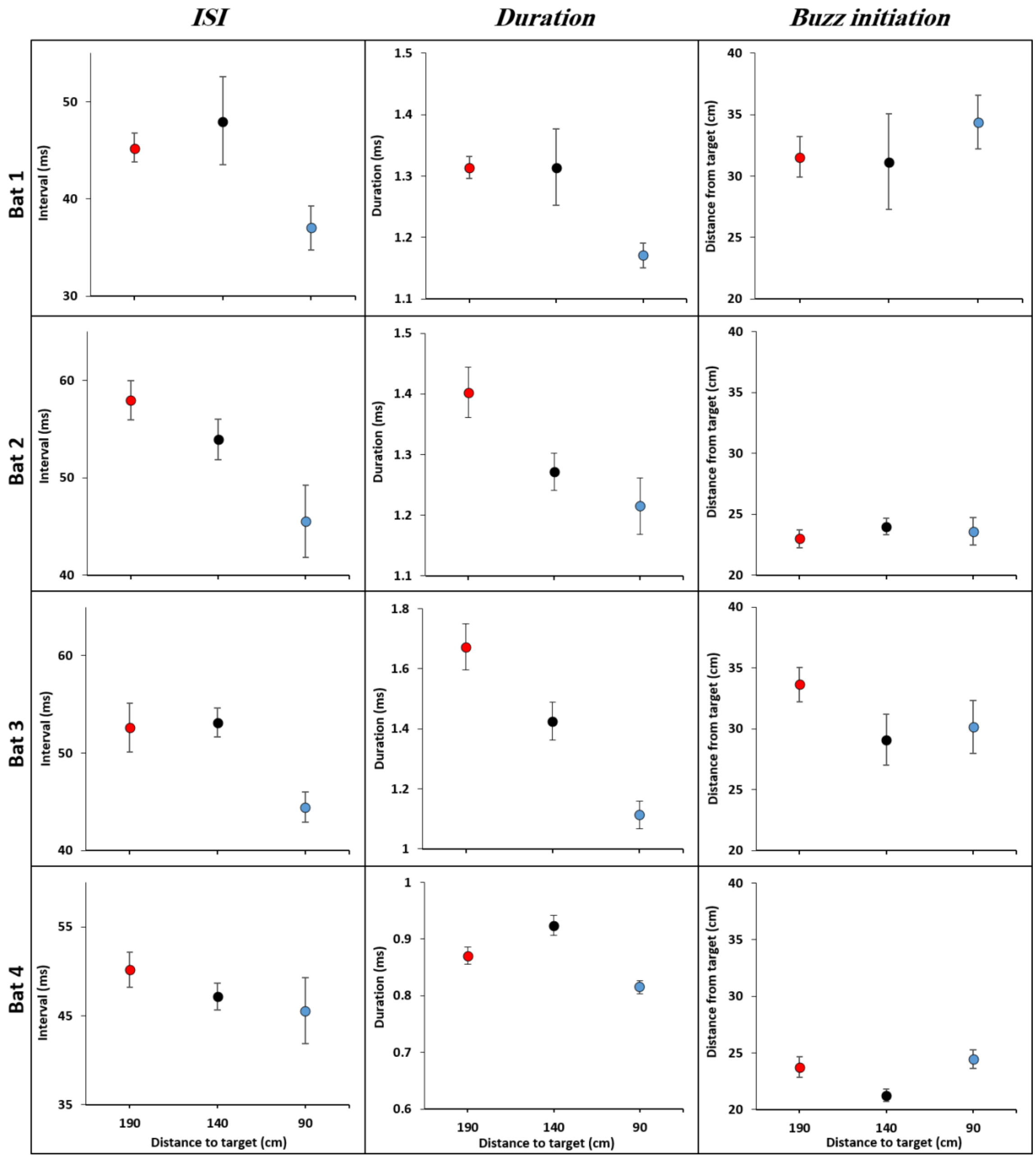

S3: Bats assess target distance before takeoff and initiate buzz at a fixed distance. For all individuals signal duration and interval between signal groups immediately before takeoff were significantly shorter when the target was closer. Buzz was initiated at a constant distance from target regardless of initial target distance. Data shown are mean ± se.

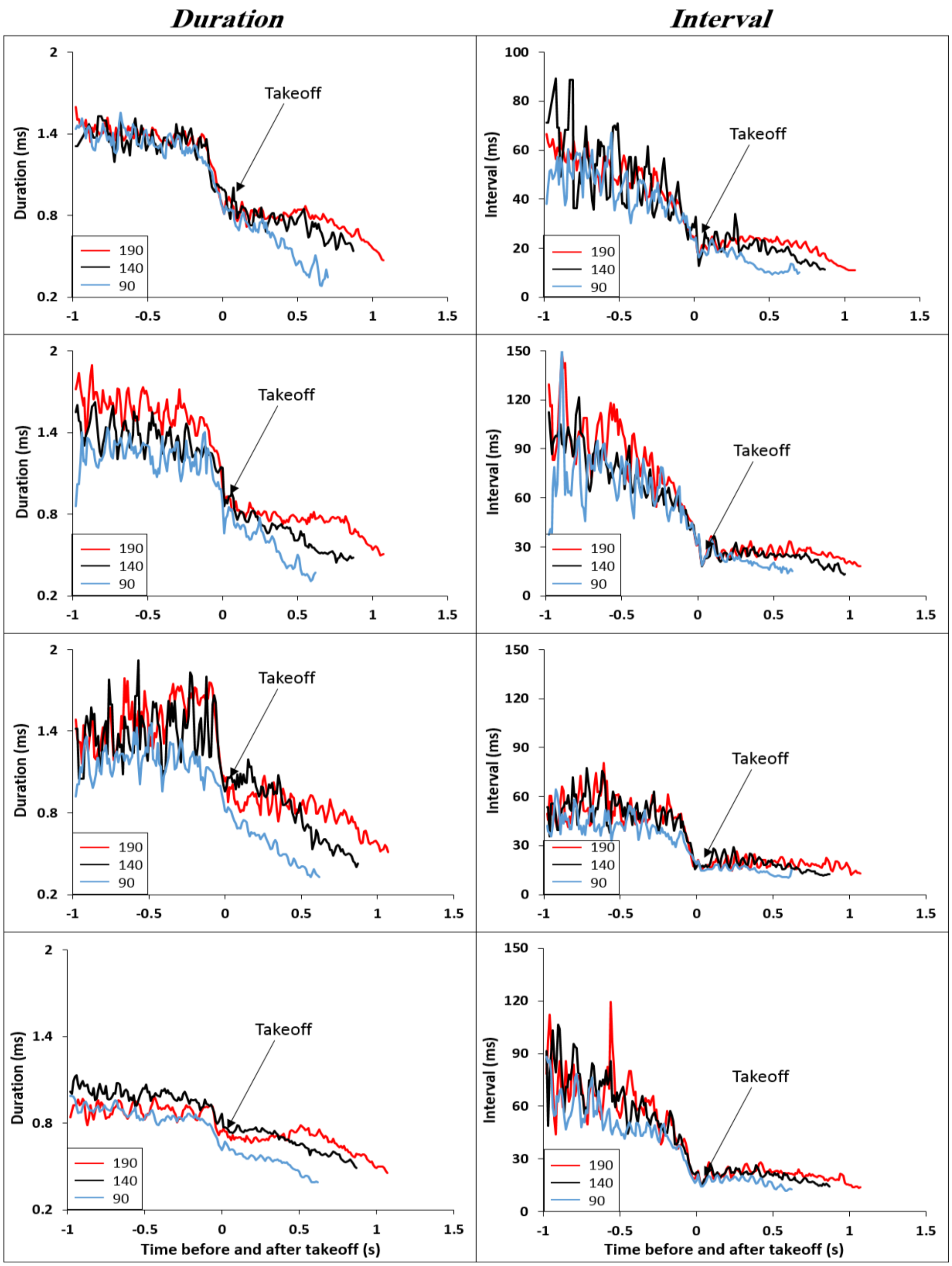

S4: Bats assess distance to target before takeoff. For all individuals, signal durations and intervals between signal groups as a function of time before and after takeoff (time point '0'). Longer signals and intervals are evident before takeoff and were probably used to assess the distance. At takeoff there is a dramatic drop in these parameters.

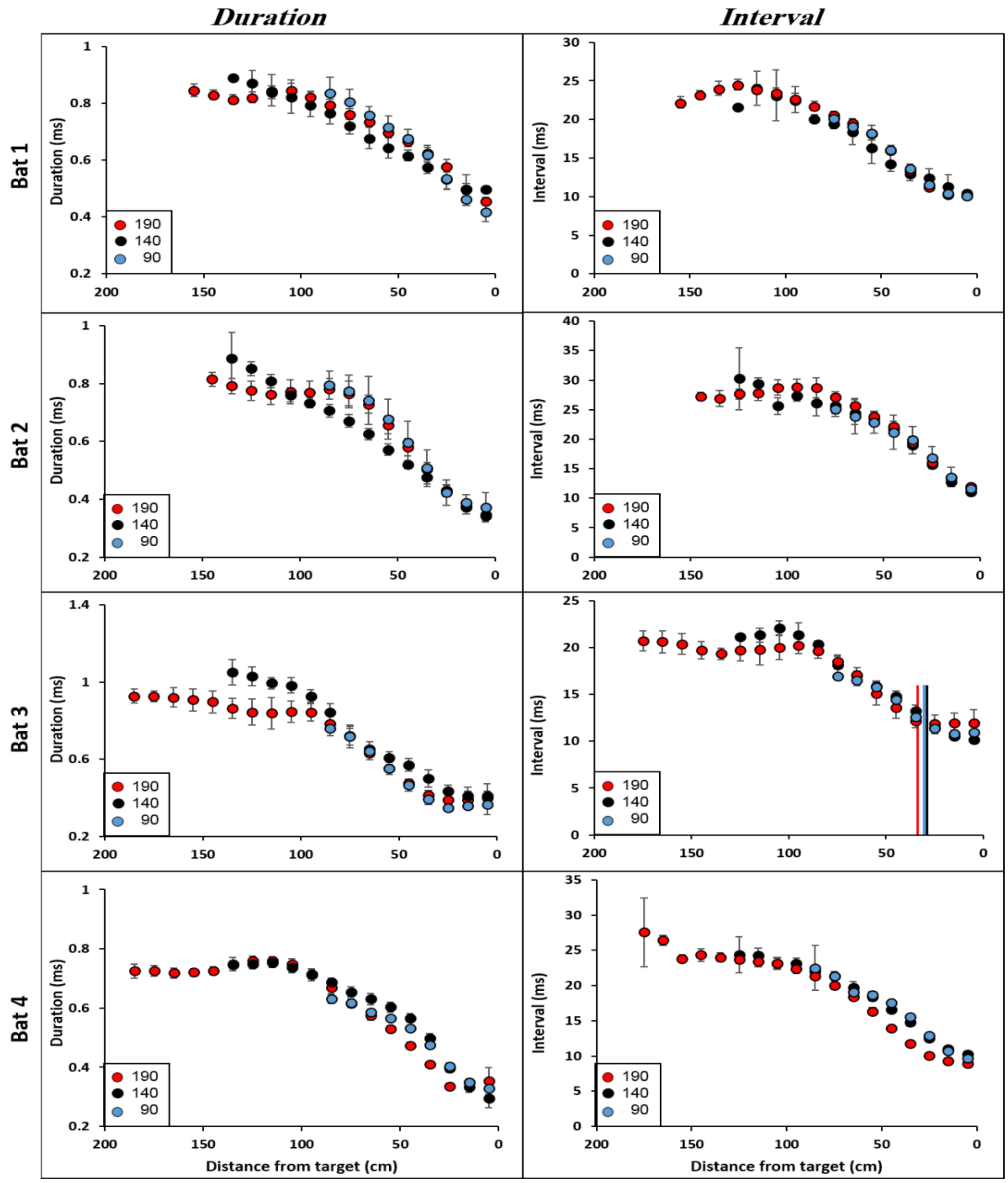

S5: Bats maintain a fixed approach phase during flight. When flying to a closer target (blue or black circles) al individuals started their approach 'in step': signal duration and interval between signal groups at takeoff were appropriate as if the bat reached this distance at flight from a greater distance. For individuals 1 & 2 flying to a target at 190cm, the first 30cm were not covered by the cameras and echolocation signals here were not synchronized to distance. Since takeoff always occurred within signal group, characterized by short inter-signal intervals, we removed the takeoff group from this analysis.

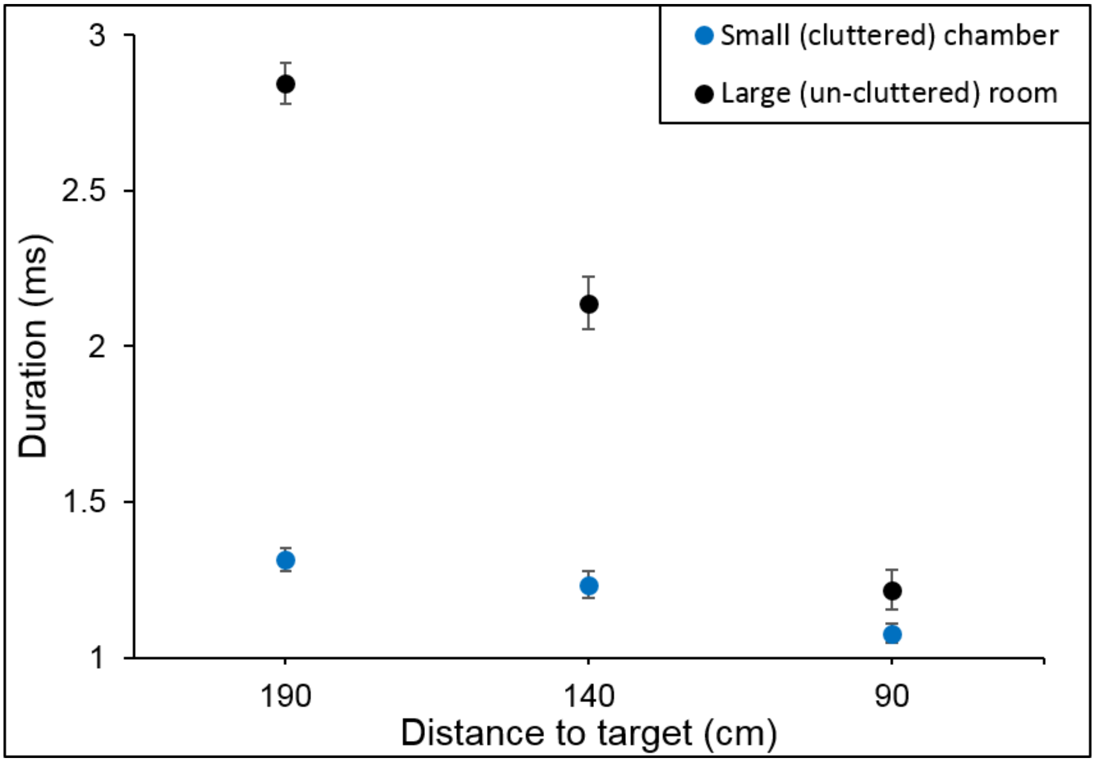

S6: clutter and background echoes influence approach sequence design. When preparing for flight in the small (i.e. cluttered) chamber (blue points) bats used much shorter signals than those produced at the same distances from target by bats flying in a large, un-cluttered room, showing acoustic environment shapes overall signal design but not target ranging behaviour (Figures 1 & 2). The difference decreased as the distance decreased and information flow-rate became the dominant factor. Values given are mean ± se.

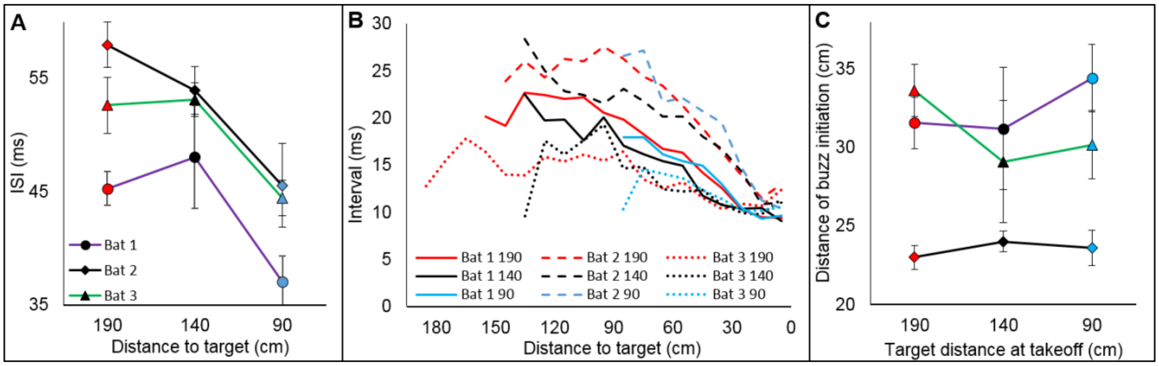

S7. Inter-individual variation. While applying the same sensorimotor adaptations, individual bats did so in slightly different strategies, e.g. bat 2 used longer intervals between signals both before (A) and during (B) flight than bats 2&3, and initiated its buzz closer to the target (C). The overall pattern though, was the same. Bat 4 was omitted from this presentation to prevent visual clutter.
